# A Comprehensive Identification and Expression Analysis of the WUSCHEL Homeobox-Containing Protein Family Reveals Their Special Role in Development and Abiotic Stress Response in *Zea mays* L.

**DOI:** 10.3390/ijms25010441

**Published:** 2023-12-28

**Authors:** Xuanxuan Chen, Yunyan Hou, Yongyan Cao, Bo Wei, Lei Gu

**Affiliations:** School of Life Sciences, Guizhou Normal University, Guiyang 550025, China; 222100100380@gznu.edu.cn (X.C.); 21010100376@gznu.edu.cn (Y.H.); 232100100386@gznu.edu.cn (Y.C.); 232100100408@gznu.edu.cn (B.W.)

**Keywords:** abiotic stress, WOX gene family, genome-wide analysis, maize, WUS homeobox

## Abstract

Maize is an important food and cash crop worldwide. The WUSCHEL (WUS)-related homeobox (WOX) transcription factor (TF) family plays a significant role in the development process and the response to abiotic stress of plants. However, few studies have been reported on the function of *WOX* genes in maize. This work, utilizing the latest maize B73 reference genome, results in the identification of 22 putative *ZmWOX* gene family members. Except for chromosome 5, the 22 *ZmWOX* genes were homogeneously distributed on the other nine chromosomes and showed three tandem duplication and 10 segmental duplication events. Based on phylogenetic characteristics, ZmWOXs are divided into three clades (e.g., WUS, intermediate, and ancient groups), and the majority of ZmWOXs in same group display similar gene and protein structures. Cross-species collinearity results indicated that some *WOX* genes might be evolutionarily conservative. The promoter region of *ZmWOX* family members is enriched in light, plant growth/hormone, and abiotic stress-responsive elements. Tissue-specific expression evaluation showed that *ZmWOX* genes might play a significant role in the occurrence of maize reproductive organs. Transcriptome data and RT-qPCR analysis further showed that six *ZmWOX* genes (e.g., *ZmWOX1*, *4*, *6*, *13*, *16*, and *18*) were positively or negatively modulated by temperature, salt, and waterlogging stresses. Moreover, two *ZmWOXs*, *ZmWOX1* and *ZmWOX18*, both were upregulated by abiotic stress. ZmWOX18 was localized in the nucleus and had transactivation activities, while ZmWOX1 was localized in both the cytoplasm and nucleus, without transactivation activity. Overall, this work offers new perspectives on the evolutionary relationships of *ZmWOX* genes and might provide a resource for further detecting the biological functions of *ZmWOXs*.

## 1. Introduction

The development of apical meristematic tissue and the differentiation of lateral organs affect the plant shape and height. The homeobox (HB) gene family is a large group of plants (containing 14 subfamilies). Mutations in these classes of genes can lead to embryonic or other periods of developmental abnormalities [1]. The WUSCHEL-related homeobox (WOX) TF gene group is among the most important HB families. The protein N-terminus of its family members contains a conserved DNA-binding region consisting of three α-helices (e.g., homeodomain and HD, including 60–66 amino acids) [2]. In addition to the conservative HD motif, the WOX protein also contains some functional domains in the C-terminal, such as the typical WUS-box structural domain (TLXLFP, seven amino acids, X indicates any amino acid), EAR motif (ERF-associated amphiphilic repression), and acidic domain [3,4]. The WOX proteins in many species may be divided into three groups based on their structural similarity: ancient, intermediate, and modern/WUS [5]. Ancient *WOX* genes are widely distributed in plants. Intermediate group members are found in vascular plants. Finally, WUS exists only in seed plants [6].

According to bioinformatics analysis, the *WOX* gene family has been identified in many plants, such as Arabidopsis (15 members) [7,8,9], melons (*Cucumis melo* L.) (11 members) [10], Sacred Lotus (*Nelumbo nucifera*) (15 members) [11], tomatoes (10 members) [12], upland cotton (40 members) [13], sunflowers (*Helianthus annuus* L.) (18 members) [14], bread wheat (14 members) [15], rice (13 members) [16,17], and sorghum (11 members) [18]. The functions of WOX in the regulation of primary and secondary growth processes of shoot apical meristem (SAM)/root apical meristem (RAM)/vascular meristems and organ (embryo and flower) development have been systematically reported in Arabidopsis [19,20]. The AtWUS protein maintains stem cell homeostasis in the SAM to ensure normal plant development [21,22,23]. Like AtSUS, AtWOX5 is essential for stem cell homeostasis in the RAM [21,24]. AtWOX3 functions in the initiation and growth of lateral organs [25]. AtWOX4 is abundantly functional with AtWOX14 and accepts the signal from the CLE41/44-PXY complex to be responsible for vascular organization [26]. AtWOX14 also enhances gibberellin (GA) synthesis to promote vascular cell development [27]. The *AtWOX13* gene participates in root and flower development [28]. In rice, the *OsWUS* gene is expressed in leaf primordium and RAM [17]. OsWOX3, which is homologous to AtWOX3, may play a significant role in the development of leaves [29]. OsWOX4 plays a crucial regulatory role in early leaf development. The knockdown of *OsWOX4* leads to leaf development defects [30]. *OsWOX11* is expressed in newborn coronal roots and subsequently in the cell division region of root meristem tissue [31].

In addition to playing a crucial regulatory role in plant development, WOX TFs could also be modulated by various abiotic stresses (e.g., drought, low temperature, and salt) [32]. In rice, OsWOX11 regulates the development process of root hair to increase rice drought tolerance [31]. Eight *OsWOX* genes, including *OsWUS*, positively responded to drought stress. For instance, *OsWOX3* and *OsWOX5* could be induced by salt, while four members of *OsWOX* respond positively to cold [29]. Some *WOX* gene family members in cotton and cucumber also respond positively or negatively to several environmental stresses [18,33]. The *OsWOX13* gene is modulated by drought, cold, and salt. Overexpression of *OsWOX13* in rice significantly enhanced plant drought stress tolerance [34]. Overexpression of *MdWOX13-1* also enhanced plant drought stress resistance by activating the reactive oxygen species scavenging system [35].

Maize is an important crop worldwide. Previous studies have shown 21 *ZmWOX* genes in maize based on ancient genome data [16]. Four *ZmWOX* genes (e.g., *ZmNS1*, *ZmNS2*, *ZmWUS1*, and *ZmWUS2*) were also involved in leaf development and promoted somatic embryogenesis in maize. Double mutants of *ZmNS1* and *ZmNS2* result in narrow leaf width but unchanged leaf length [36]. Both *ZmWUS1* and *ZmWUS2* are homologous to *AtWUS*. At the same time, the expression patterns of these two genes have significant differences; the transcript of *ZmWUS1* is in dynamic change during the nutrient growth stage, and the *ZmWUS2* gene is specifically expressed in young leaf primordia [37]. Meanwhile, co-overexpression of *ZmWUS2* and *BBM* genes in maize notably enhances somatic embryogenesis and shortens the time of genetic transformation [38]. Despite the above findings, the function of *ZmWOX* genes in maize development and the abiotic stress response is largely unknown.

In this work, we identified 22 *WOX* genes in maize and conducted a comprehensive analysis of these genes according to the newly assembled maize B73 reference genome (Zm-B73-REFERENCE-NAM-5.0). The expression level of *ZmWOXs* under normal and abiotic conditions was also explored. Meanwhile, the subcellular localization and transcriptional activation activity of ZmWOX1 and ZmWOX18 were analyzed. This study provides new information about the evolutionary relationships of the *ZmWOX* genes and may help further understand the possible functions of ZmWOXs.

## 2. Results

### 2.1. Identification and Characterization of ZmWOX Genes

A 2010 study reported a maize genome containing 21 *WOX* genes [16]. In this study, based on a new version of the maize B73 genome (NAM-5.0), a homology search was performed using known AtWOX and OsWOX sequences [15,16] as detectors. After removing duplicates and confirming the conservative HD domain, 22 putative *WOX* genes were identified in maize (Table 1). The detailed location site of each *ZmWOX* gene was obtained after searching the genome (Supplementary Table S1). The 22 *ZmWOX* genes were labeled *ZmWOX1* to *ZmWOX22* according to their location in the chromosomal region (Supplementary Table S1). Except for chromosome 5, the other nine chromosomes all contained *ZmWOXs*. Chromosome 8 had the most *ZmWOX* genes (five members), followed by four members located on chromosome 3, while chromosomes 1, 7, and 9 only included one *ZmWOX* gene, respectively (Supplementary Table S1). The coding sequence (CDS) of *ZmWOXs* ranged from 252 bp (*ZmWOX8*) to 1551 bp (*ZmWOX7*), and 22 ZmWOX proteins contained 83–516 amino acids (aa) (average: 287 aa) (Table 1). The isoelectric points (PI) and molecular weights of the ZmWOXs ranged from 5.56 to 10.52 (average: 8.00) and 10.01 to 53.26 kDa (average: 30.67), respectively (Table 1). According to the predicted results of the website, most ZmWOX proteins (17 members) were distributed in the nucleus (Table 1), consistent with the localization of transcription factors (TF). The other five members were distributed in the chloroplast (three members) and mitochondrion (two members), respectively (Table 1). It may be responsible for the transcriptional regulation of genes in plastids.

### 2.2. Phylogenetic Analysis of ZmWOX Proteins

**Figure 1:**
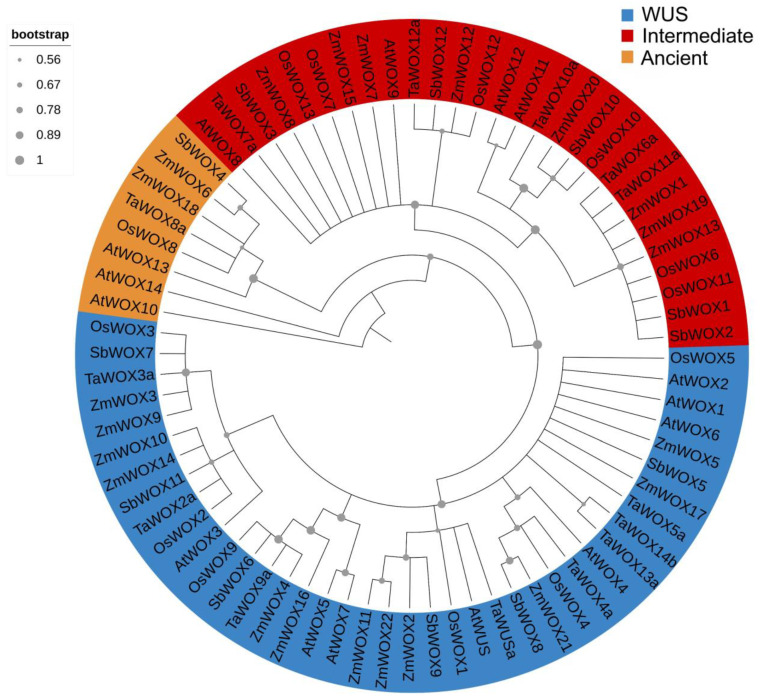
Phylogenetic analysis of WOX homologs in maize (Zm), sorghum (Sb), rice (Os), wheat (Ta), and Arabidopsis (At). The unrooted neighbor-joining phylogenetic tree building by MEGA 7.0 (10,000 bootstrap replicates) was divided into three groups. Each branch is represented by specific colors.

The Arabidopsis genome includes 15 *WOXs*, and the protein sequences of these genes can be classified into three groups (e.g., modern/WUS, intermediate, and ancient) according to evolutionary relationships [39]. We constructed a phylogenetic tree using the protein sequences of known WOXs in sorghum [16] (12 WOX members in sorghum, Supplementary Table S1), rice (Supplementary Table S2) [17], wheat (Supplementary Table S2) [15], Arabidopsis (Supplementary Table S2) [8], and the newly identified ZmWOXs to understand the evolutionary relationships of *WOX* genes in maize. As shown in Figure 1, 76 WOX proteins from five plants could also be grouped into three clades (e.g., WUS, intermediate, and ancient), each containing 40, 28, and 8 *WOX* genes, respectively (Figure 1). For the maize WOX family, twelve ZmWOXs (e.g., ZmWOX2–5, 9–11, 14, 16, 17, 21, 22) belong to the WUS group, eight ZmWOX proteins (e.g., WOX1, 7, 8, 12, 13, 15, 19, 20) are in the intermediate clade, and only two ZmWOXs (e.g., ZmWOX6 and 18) are subdivided into the ancient group (Figure 1). The WOX proteins in each group were all from five plants, revealing that the *WOX* genes in these species may experience similar evolutionary models (Figure 1). Meanwhile, *ZmWOX* genes in all clades were more closely related to *SbWOX* genes than to the other *WOXs* (Figure 1), indicating maize and sorghum are evolutionary orthologous relationships.

### 2.3. Structural Analysis of ZmWOX Genes

**Figure 2:**
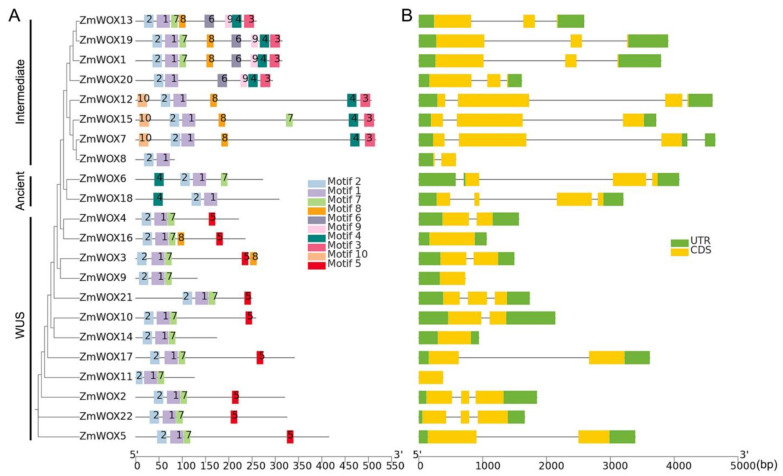
Gene structure and conserved protein motifs of *ZmWOXs*. The clustering of *ZmWOX* genes was based on the phylogenetic tree shown in Figure 1. (**A**) Distribution of 10 conserved motifs (differently colored boxes) in ZmWOX proteins. The common sequences of motifs 1–10 are shown in Figure S1. (**B**) Exon–intron structure of 22 *ZmWOXs*. Introns as lines.

MEME and TBtools were used to analyze the protein motifs and gene structure of *ZmWOXs*. According to the MEME-detected results, 10 conserved motifs were in 22 ZmWOXs. The conserved HD domain (e.g., motifs 1 and 2, Helix1-Loop-Helix2-Turn-Helix3) (Figure S1) was present in all ZmWOXs (Figure 2A). Except for motifs 1 and 2, some motifs were only present in special groups, reflecting the diversity of ZmWOXs. The intermediate clade members contained motifs 3, 4, 6, 9, and 10. Meanwhile, both members included motifs 3–4 in the C terminal, except for ZmWOX8 (short sequence) (Figure 2A). ZmWOX6 and ZmWOX18 (ancient members) also contained motif 4 at the N-terminus (Figure 2A). In Arabidopsis, WUS subfamily members contained two conserved domains: WUS box and EAR-like. All members contained the WUS box, while the EAR motif was found only in AtWUS [39]. As shown in Figure 2A, except for ZmWOX9, 11, and 14 (short sequence), motif 5 (WUS box, TLXLFP, Figure S1) was identified in all maize WUS members. We also found the EAR domain in ZmWOX2 (ZmWUS1) and ZmWOX22 (ZmWUS2) (Figure S2).

We analyzed the exon–intron distribution of *ZmWOX* genes using TBtools. Except for *ZmWOX9* and *ZmWOX11*, which had only one exon and no intron, all *ZmWOXs* contained at least two exons and one intron (Figure 2B). The exon and intron lengths were similar in the same clade. Still, it displayed wide variation between the different subfamilies, mainly due to the differences in the intron sequence (Figure 2B). The intermediate members contained longer exon sequences than the WUS and ancient clades (Figure 2B), leading to longer protein sequences (Figure 2A). Overall, these results revealed that the same clade of *ZmWOX* genes shows less variation in gene and protein structure and may display the same functions.

### 2.4. Gene Duplication and Synteny Analysis of the ZmWOX Genes

**Figure 3:**
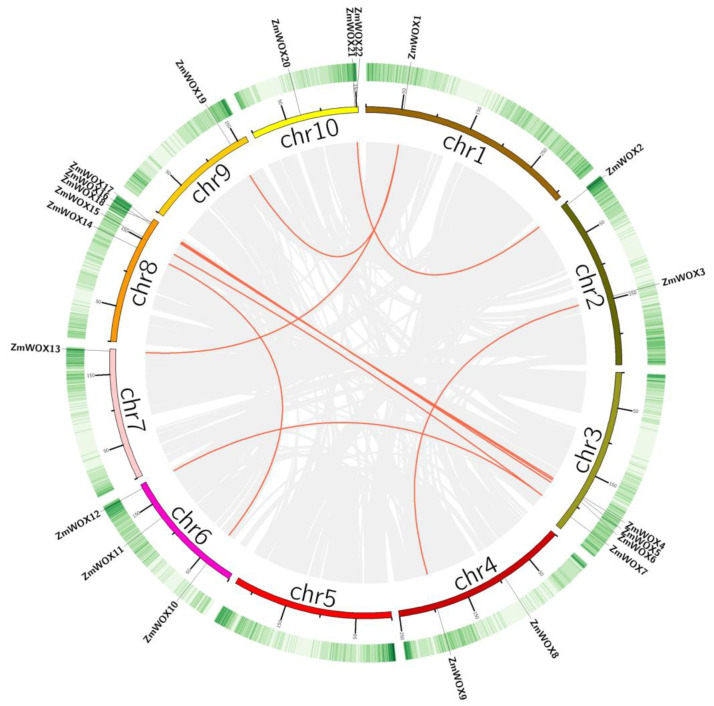
Gene duplication events for *ZmWOX* genes in the maize genome. Schematic diagram of the relationship of *ZmWOX* genes between chromosomes. The chromosome locations of each *ZmWOX* gene are shown in the figure. The heatmap in the outside circles indicates chromosome gene density. The gray lines on the inside indicate collinear gene pairs in the maize genome. The collinear *ZmWOX* genes are connected through red line.

Except for chromosome 5, the 22 *ZmWOXs* were widely distributed in the remaining nine chromosomes (Supplementary Table S1 and Figure 3). Some *ZmWOX* genes were located close together and formed three tandem duplication events (*ZmWOX4*/*5*/*6*, *ZmWOX16*/*17*/*18*, and *ZmWOX21*/*22*) (Figure 3). According to the gene duplication events analysis, except for three tandem duplication events (Figure 3), 10 pairs of segmental duplication (homologous) in *ZmWOX* genes were also identified in the maize genome (Figure 3). Among these 10 pairs, five belonged to the WUS family (e.g., *ZmWOX2*/*22*, *ZmWOX3*/*9*, *ZmWOX4*/*16*, *ZmWOX5*/*17*, and *ZmWOX10*/*14*), four in the intermediate clade (e.g., *ZmWOX1*/*13*, *ZmWOX1*/*19*, *ZmWOX7*/*12*, and *ZmWOX7*/*15*), and only *ZmWOX6*/*18* in the ancient group (Figure 3). These results indicate that segmental duplication may be the main force driving the extension of the *ZmWOXs* family.

**Figure 4:**
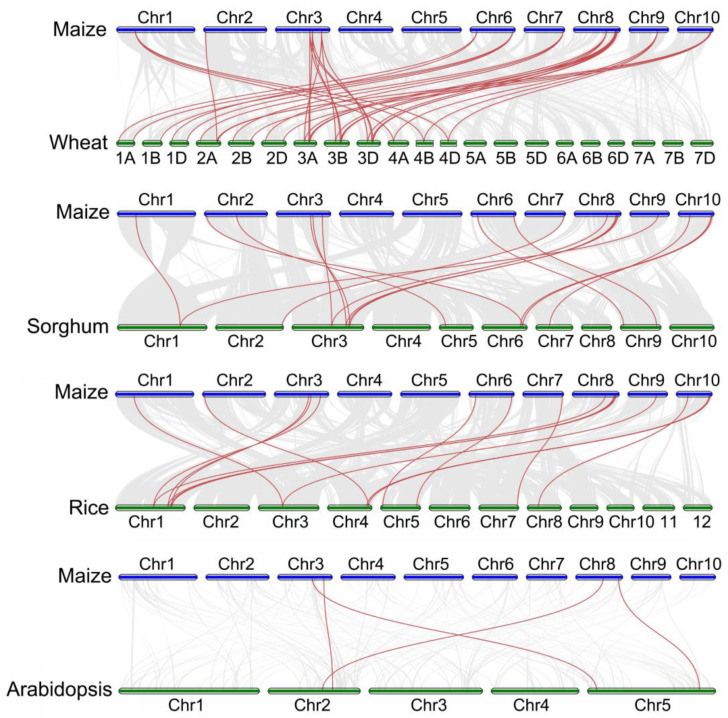
The collinearity diagram of *WOX* genes between maize, wheat, sorghum, rice, and Arabidopsis. The gray line in the background represents the collinear gene pairs between the two species, and the *WOX* collinear genes are connected by red lines.

A collinear analysis between maize and the other four species (i.e., wheat, sorghum, rice, and Arabidopsis) was constructed to further understand the evolutionary mechanism of the *ZmWOX* genes. As shown in Figure 4, an amount of 40, 17, 16, and four collinear gene pairs (a total of 77) were identified between maize and wheat, maize and sorghum, maize and rice, and maize and Arabidopsis, respectively. This result indicated that the amount of *WOX* homologous genes is high in crops and that paralogous homologous genes may play a significant role in the WOX family evolution process. *ZmWOX4* (Chr 3), *7* (Chr 3), *14* (Chr 8), and *17* (Chr 8) showed homologous pairs in all five plants (Figure 4), indicating that these four genes may have existed before the evolution of these five plant species. The results suggest that the *ZmWOX* family genes may have similar functions to the WOXs of other species.

### 2.5. The Cis-Element Analysis of the ZmWOX Promoter

**Figure 5:**
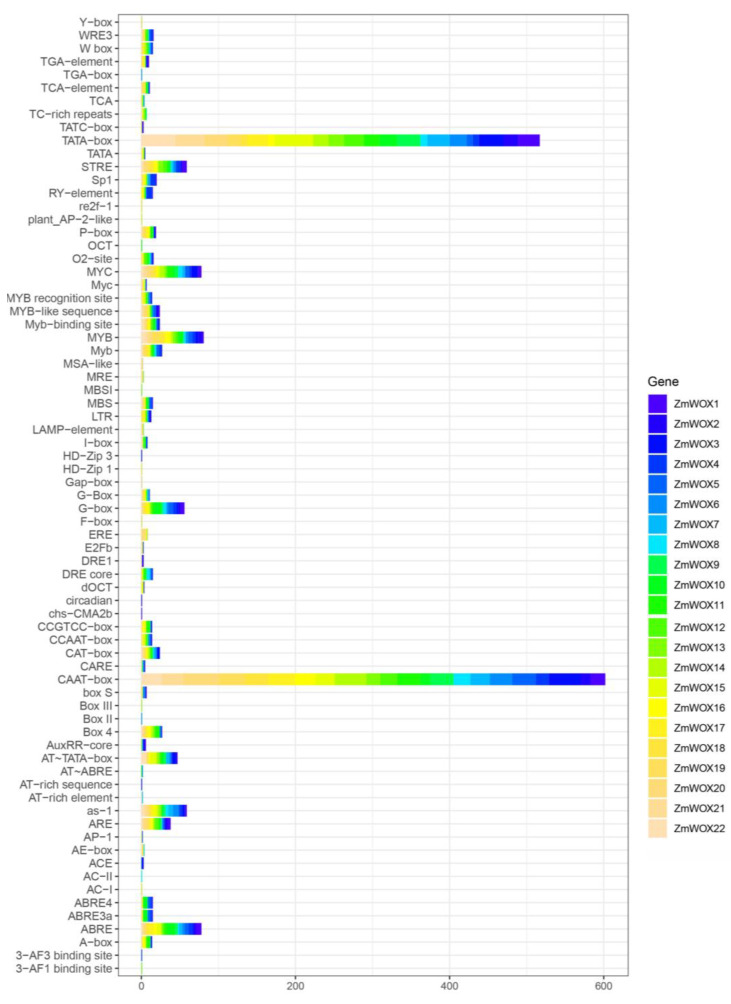
The *cis*-element analysis in promoter regions of *ZmWOX* genes. A promoter region of about 2 kb upstream of the *ZmWOX* genes was downloaded from the genome for analysis. Different colors represent different genes. The *cis*-element names and counts are listed in Supplementary Table S3.

A 2 kb sequence upstream of the start codon of each gene was downloaded from the maize genome NAM 5.0 and submitted to the online PlantCARE database to detect the diversity of *cis*-elements present in the promoter regions of the *ZmWOX* genes. As shown in Figure 5 and Supplementary Table S3, except for the TATA box and CAAT box, 1037 elements were found in the promoter of 22 *ZmWOXs*. These elements could be divided into four classes: light responsive (e.g., Box 4, G-box, I-box, AE-box, MRE, Sp1, and Gap-box), hormones responsive (e.g., ABA-responsive elements (ABRE), TCA-element, TGA-element, TATC-box, P-box, TCA, ERE, and AuxRR-core), plant growth (e.g., ARE, CAT-box, circadian, MBSI, MSA-like, O2-site, AT-rich element, and RY-element), and stress-responsive elements (e.g., STRE, Myc, MYB site, W box, MBS, LTR, drought-responsive elements (DRE) core, and TC-rich repeats). Some elements were widely located in the *ZmWOXs* promoter, such as ABRE (contained in 20 members), MYB/MYC binding site elements related to abiotic stress and plant growth (contained in all members), and STRE elements (18 *ZmWOXs*) (Figure 5 and Supplementary Table S3). These results suggest that the *ZmWOX* genes might be involved in stress resistance and the maize growth/hormone pathway.

### 2.6. Expression Patterns of ZmWOX Genes in Different Maize Tissues

**Figure 6:**
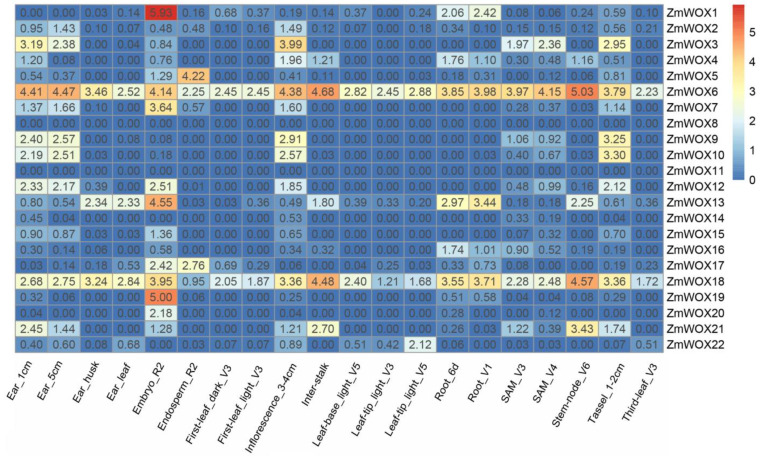
The heatmap of *ZmWOX* gene expression levels in different maize tissue. The heatmap construction is based on the fragments per kilobase per million mapped reads (FPKM) values of published RNA-seq data (Supplementary Table S4). The name of the tissues is labeled in the Figure. SAM: shoot apical meristem.

Published RNA-seq datasets [40] (Supplementary Table S4) from 15 maize tissues with varied developmental periods were used to explore the expression patterns of *ZmWOX* genes in the maize growth and development stage. As shown in Figure 6, except for *ZmWOX8* and *ZmWOX11*, all tissues had no expression. The other 20 genes showed high expression levels in specific tissues, especially for *ZmWOX6* and *18* displayed high expression levels in all tissues. Meanwhile, *ZmWOXs* in reproductive organs (e.g., ear, embryo, inflorescence, and tassel) showed relatively higher transcript amounts compared to nutrient tissue (e.g., leaf, stem, and root) (Figure 6). Except for *ZmWOX6*, *16*, *18*, *21*, and *22*, the peak expression level of the other genes was in all reproductive tissues (Figure 6). For example, the expression of *ZmWOX1* in embryo was almost triple as high as that in the roots (Figure 6). These results indicate that *ZmWOXs* might play an important role in maize development, especially in the occurrence of reproductive organs.

### 2.7. Expression Patterns of ZmWOXs under Abiotic Stress Conditions

**Figure 7:**
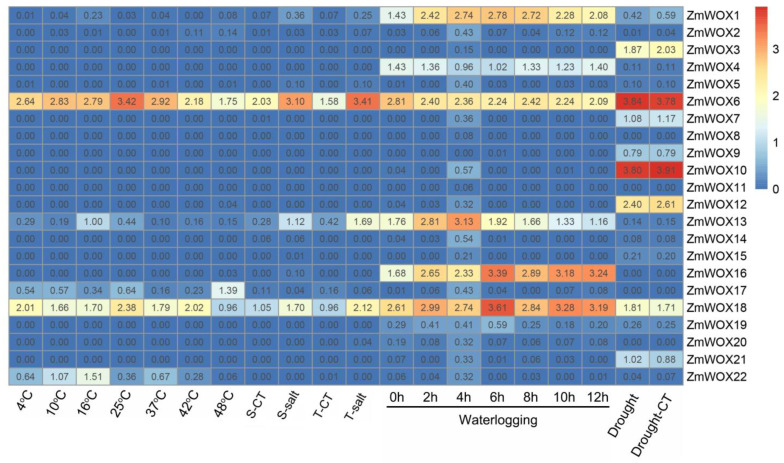
Heatmap of *ZmWOX* gene expression levels under temperature, salt, waterlogging, and drought stresses. Different stresses are labeled in the figure. The data (except drought stress) represent the mean value of three replicates of published transcriptomic data (Supplementary Table S5). For salt stress, ‘S’ indicates a salt-sensitive maize-inbred line (L29). ‘T’ indicates a salt-tolerant maize-inbred line (L87). Heatmap construction is based on the fragments per kilobase per million mapped reads (FPKM) values.

We used the published transcriptome data about drought [41] (Supplementary Table S5), salt [42] (Supplementary Table S5), temperature [43] (Supplementary Table S5), and waterlogging [44] (Supplementary Table S5) stresses to analyze the expression patterns of *ZmWOX* genes under abiotic stresses. As shown in Figure 7, *ZmWOX6* was downregulated by heat and cold treatments. *ZmWOX13* and *ZmWOX22* (*ZmWUS2*) positively responded to cold stress, while cold stress inhibited *ZmWOX17*. The other members of *ZmWOXs* did not seem to be influenced by temperature stress (Figure 7). For salt stress, whether in salt-stress-sensitive or salt-tolerant maize lines, *ZmWOX1*, *ZmWOX2*, *ZmWOX6*, *ZmWOX13*, and *ZmWOX18* were positively modulated by salt stress (Figure 7). For waterlogging maize root stress, except for *ZmWOX4* and *6* (downregulated by waterlogging), the other 20 *ZmWOXs* were all induced by the waterlogging stress (Figure 7), implying that these genes might show a key function in maize root response to low oxygen stress. Unlike the above three abiotic stresses, the *ZmWOXs* did not seem to respond to drought stress (Figure 7), even though some of the promoters of these genes contained DRE (Figure 5 and Supplementary Table S3).

### 2.8. RT-qPCR Analysis of ZmWOX Genes under Abiotic Stresses

**Figure 8:**
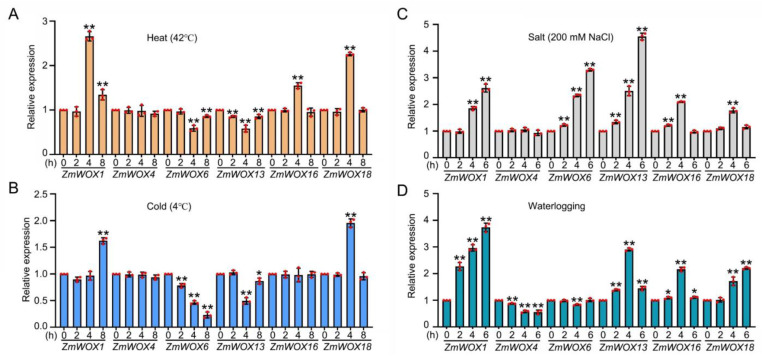
RT-qPCR analysis of the expression of *ZmWOX1*, *4*, *6*, *13*, *16*, and *18* genes in maize seedlings under heat (**A**), cold (**B**), 200 mM NaCl (**C**), and waterlogging (**D**) stresses. Treatment times and conditions are labeled in the figure. Values are means ± SD; *n* = 3. ** *p* < 0.01, * *p* < 0.05 compared with 0 h (Student’s *t*-test).

*ZmWOX1*, *4*, *6*, *13*, *16*, and *18* were simultaneously positively or negatively modulated by temperature, salt, and waterlogging stresses (Figure 7). These genes were chosen to further explore the expression levels of *ZmWOX* genes in heat, cold, salt, and waterlogging stresses in treated maize lines using RT-qPCR. *ZmWOX1*, *ZmWOX16*, and *ZmWOX18* were induced by heat shock, and *ZmWOX6* and *13* were downregulated by heat and cold stress. In contrast, *ZmWOX4* did not respond to temperature stress (Figure 8). For salt stress (200 mM NaCl), except for *ZmWOX4* (no change), the transcript levels of the other five genes were upregulated by salt stress (Figure 8). Although *ZmWOX4* was not responsible for temperature and salt stresses, the expression level of this gene was reduced under waterlogging treatment (Figure 8). Low oxygen stress also induced the other four genes (*ZmWOX1*, *13*, *16*, and *18*) or downregulated *ZmWOX6*. Overall, six *ZmWOX* genes displayed different expression patterns in abiotic stresses.

### 2.9. Subcellular Localization and Transactivation Activity Assays of ZmWOX1 and 18

**Figure 9:**
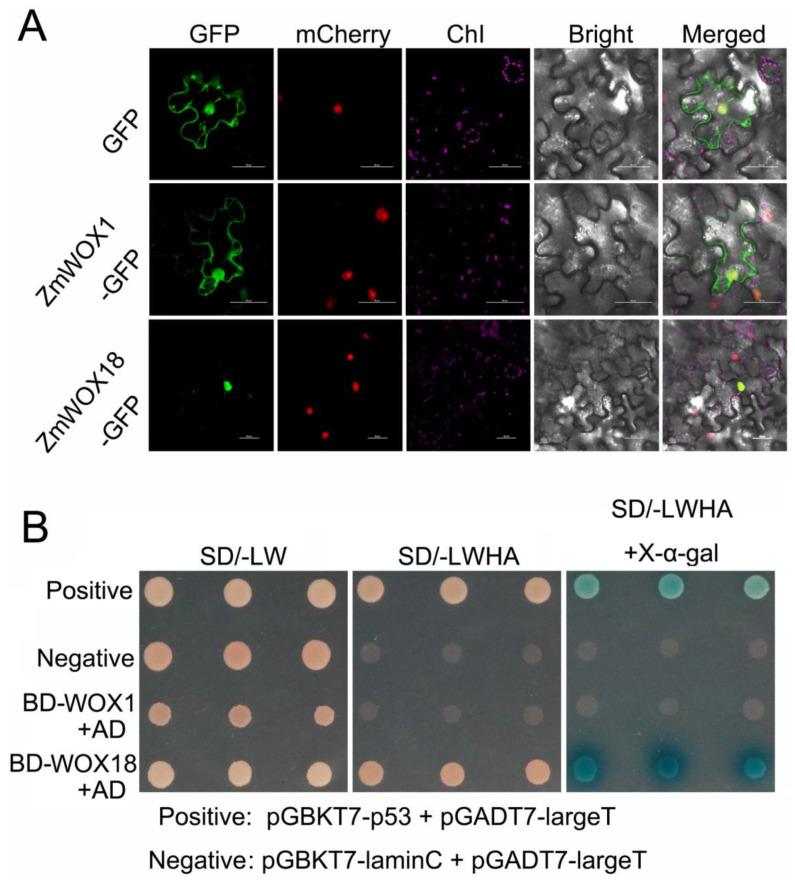
Subcellular localization and transactivation activity assays of ZmWOX1 and ZmWOX18. (**A**) Subcellular localization of ZmWOX1 and ZmWOX18. The GFP or 35S∷ZmWOX1/18-GFP fusion expression vectors were cotransformed with mCherry (nuclear marker) into tobacco leaves using an Agrobacterium-mediated method. The GFP, mCherry, and chlorophyll autofluorescence (ChI) signals are labeled green, red, and purple, respectively. The empty GFP vector was used as a control. GFP: green fluorescent protein. Bars = 50 μm. (**B**) Transcription activation analysis of ZmWOX1 and ZmWOX18 in yeast. Full-length *ZmWOX1* or *ZmWOX18* was fused with the GAL4-DNA BD in the pGBKT7 vector (BD-WOX1 or BD-WOX18). BD-WOX1/18 and pGADT7(AD) were cotransformed into yeast Y2H gold. The yeast cells were cultured on a synthetic dextrose medium (SD) lacking tryptophan and leucine (SD-LW), then transferred to an SD medium without tryptophan, leucine, histidine, and adenine (SD-LWHA, containing or not containing X-α-gal) to detect interactions. The positive or negative controls are labeled in the figure, respectively.

*ZmWOX1* and *ZmWOX18*, exhibiting positive response patterns under RT-qPCR analysis (Figure 8), were chosen to explore subcellular localization and transactivation activity to further determine the functions of *ZmWOX* genes. The 35S∷ZmWOX1/18-GFP and GFP empty vector (control) were cotransformed with the nucleus marker vector (mCherry) into tobacco leaves using the Agrobacterium-mediated method. The fluorescence signals of GFP and ZmWOX1-GFP were distributed in the cell membrane, cytoplasm, and nucleus. In contrast, the fluorescence signal of the ZmWOX18-GFP was only distributed in the nucleus, indicating that ZmWOX18 is localized in the nucleus, and ZmWOX1 was widely distributed in cells (Figure 9A). The transactivation activities of ZmWOX1 and ZmWOX18 were assessed using the Y2H yeast system. Like the positive control, yeast cells carrying pGBKT7-ZmWOX18 (BD-WOX18) and pGADT7 empty vectors grew well on an SD medium without tryptophan, leucine, histidine, and adenine (SD/-Trp/-Leu/-Ade/-His). They could show a blue color after adding X-α-gal. In contrast, the negative control and BD-WOX1+AD groups did not survive (Figure 9B). This result revealed that ZmWOX18 had transcriptional activity in yeast, while ZmWOX1 showed no transcriptional activity.

## 3. Discussion

*WOX* genes play an important role in various periods of plant development and environmental stress response [5,16,18,45]. Previous studies have reported 21 *WOX* genes in maize [16], named *ZmWOX2A*, *2B*, *3A*, *3B*, *4*, *5A*, *5B*, *9A*, *9B*, *9C*, *11*, *12A*, *12B*, *13A-D*, *ZmNS1*, *ZmNS2*, *ZmWUS1*, and *ZmWUS2*. Based on the newly released and assembled maize genome, this work identified 22 *ZmWOX* family members and renamed these 22 genes according to their chromosomal locations (Supplementary Table S1). After searching the NCBI database and comparing with our characterization results, we found *ZmWOX12A* and *12B*, *ZmWOX13A* and *ZmWOX13D*, and *ZmWOX13B* and *ZmWOX13C* were the same gene, and named them *ZmWOX13*, *ZmWOX6*, and *ZmWOX18* in this study, respectively. The other reported *ZmWOX* genes, including *ZmWOX2A*, *2B*, *3A*, *3B*, *4*, *5A*, *5B*, *9A*, *9B*, *9C*, *11*, *ZmNS1*, *ZmNS2*, *ZmWUS1*, and *ZmWUS2* correspond, respectively, to *ZmWOX5*, *17*, *10*, *14*, *21*, *16*, *4*, *15*, *7*, *12*, *20*, *3*, *9*, *2*, *22* identified in this work (Table 1). *ZmWOX1*, *8*, *11*, and *19*, obtained in the recent genome (Table 1), are the new WOX members, not previously reported.

The 22 *ZmWOX* genes were widely distributed on the genome except chromosome 5 (Supplementary Table S1 and Figure 3). Gene duplication (containing tandem and segmental events) is a broad phenomenon in the evolution of plant gene family members [46]. In this work, three tandem and 10 segment events were identified in *ZmWOX* genes (Figure 3), indicating that the relatively high-segmental duplications in the maize WOX gene family might be the main driving forces for the expansion of *ZmWOXs*. In other species, such as sunflower [14], melon [10], cotton [13], and Sacred Lotus [11], the main driving forces for increasing the *WOX* members are also segmental duplications, indicating the *WOX* family in plants might prefer using segmental duplications in the evolutionary process. Notably, *ZmWOX8*, *11*, *20*, and *21* were not included in gene duplication events (Figure 3), although *ZmWOX20*/*21* showed high expression levels in embryo, stem, and submergence stress (Figure 6 and Figure 7). *ZmWOX8* and *11* have almost no expression in maize development and abiotic stress response (Figure 6 and Figure 7), suggesting that these two genes might be the ancient and silenced genes. Evolutionary relationship analysis using WOX protein full-length sequences of maize and four other plant species (e.g., sorghum, wheat, rice, and Arabidopsis) indicated that the maize WOX family was divided into three clades (e.g., WUS, intermediate, and ancient) (Figure 1). This condition was consistent with the classification of the WOX family in other species [5]. In addition to the evolutionary tree, we also used cross-species collinearity analysis to explore the relationship of the *WOX* family. A high homology was observed between maize and the other three Poaceae plants (Figure 4). Although there was a low collinear relationship between maize and Arabidopsis, there were also two pairs of homologous genes in these two species (Figure 4). These results indicate that *WOX* genes may undergo conservative evolutionary progress in plants and might have similar biological functions among different species. Notably, *ZmWOX8* and *9* on chromosome 4, without relationships with the other four plants (Figure 4), imply that some *WOXs* might have special features only displayed in their species.

All ZmWOXs containing the typical HD conserve domain (motifs 1 and 2) (Figure 2) and members in the same groups display similar gene and protein structures (Figure 1 and Figure 2). This reveals that different group members might show different functionalities. In other plants, *WOX* members in the same evolutionary group also display similar gene and protein structures, though with greater structural differences in different groups [10,11,13,14], suggesting that structural differences may account for functional differences of *WOX* genes in the same or different species.

In plants, the *WOX* genes mainly participate in plant growth and development, for example, in the maintenance and stabilization of SAM [23], RAM [47], inflorescence [48], and vascular [27] tissues. We found many *cis*-acting elements related to light, development, hormones, and abiotic stress response located in the promoter region of *ZmWOX* genes (Figure 5 and Supplementary Table S3). Further analysis of the published RNA-seq data about different maize tissues suggests that *ZmWOX* genes might be involved in nutrition and reproductive organ development (Figure 6). Two maize *WOX* genes, *ZmWOX6* and *18*, showed wide expression patterns in testing tissues, indicating their important role in maize development. Until now, four *ZmWOX* genes (e.g., *ZmNS1*, *ZmNS2*, *ZmWUS1*, and *ZmWUS2*, named in this work as *ZmWOX3*, *9*, *2*, and *22*, respectively) have been reported. Double mutants of *ZmNS1* and *ZmNS2* caused maize leaves to become narrow, but single mutants displayed no obvious difference [36,49]. This indicated the functional redundancy of *ZmWOX3* and *9*. Both two genes were highly expressed in inflorescence (Figure 6), implying that they may influence the reproductive process of maize. In Arabidopsis, the CLAVATA-WUSCHEL (CLV-WUS) model elaborates the regulation of division and differentiation of SAM [50]. *ZmWUS1* and *2* are homologous *AtWUS* gene but showed different expression patterns [37]. Our study indicated *ZmWUS1* (*ZmWOX2*) mainly expressed in the ear and inflorescence (Figure 6). At the same time, *ZmWUS2* (*ZmWOX22*) showed high transcript levels in leaf tips (Figure 6), suggesting that *ZmWUS1* and *ZmWUS2* might be functionally differentiated. A recent study indicated that overexpression of *TaWOX5* (homologous with *AtWUS*) in wheat could overcome the defects of genetic transformation relay on the genotype [51]. The co-overexpression of *ZmWUS2* and *BBM* genes in maize also improved genetic transformation efficiency [38]. In addition to being responsible for growth and development, *WOX* genes also respond to abiotic stress [32]. Except for *ZmWOX4* and *ZmWOX6*, the other 20 *WOX* genes were positively modulated by waterlogging stress (Figure 7), suggesting that *ZmWOXs* may be involved in the morphological reconstruction of roots under hypoxic stress. Further RT-qPCR analysis indicated *ZmWOX1*, *4*, *6*, *13*, *16*, and *18* were positively or negatively modulated by abiotic stress (e.g., temperature, salt, and waterlogging) (Figure 8), indicating that *ZmWOXs* might also play a significant role in abiotic stress.

*ZmWOX1* and *ZmWOX18* were positively modulated by heat, cold, salt, and waterlogging stress (Figure 8). We further explored the localization and transactivation activity of ZmWOX1 and ZmWOX18. ZmWOX18 was localized in the nucleus, while ZmWOX1 was present in both the nucleus and cytoplasm (Figure 9A). ZmWOX18 had transactivation activity in yeast, but ZmWOX1 seemed unable to activate downstream genes (Figure 9B). Although *ZmWOX1* and *ZmWOX18* are positively regulated by abiotic stress signals, ZmWOX1 and ZmWOX18 may play opposite roles in maize stress responses or even act reciprocally to obstruct the functioning of each other. However, this hypothesis needs to be further studied by using transgenic maize. Meanwhile, due to *ZmWOX18* being induced by many abiotic stresses (Figure 8), and ZmWOX18 exhibited activating activity (Figure 9B). It might be a useful gene for agricultural practices for breeding stress-resilient maize varieties. Our findings may offer valuable information for future crop improvement strategies.

## 4. Materials and Methods

### 4.1. Plant Materials and Stress Treatments

A maize B73 inbred line was used in this work. Surface sterilized B73 seeds were planted in soil and grown under a 16/8 h day/night photoperiod at 25 °C in a greenhouse. V3-stage maize seedlings were used to assess abiotic stress. The seedlings were placed in a 42 °C or 4 °C growth chamber for either heat or cold stress for 0, 2, 4, or 8 h [52]. The seedlings were transferred to a water solution containing 200 mM NaCl for 0, 2, 4, or 6 h for salt stress. The roots were collected for waterlogging by maintaining a 2 cm water layer above the first leaf for 0, 2, 4, or 6 h. There were three replicates for each treatment and three pots of seedlings for each replicate. The maize leaves (e.g., heat, cold, and salt) or roots (waterlogging) were harvested after stress treatments and then immediately frozen in liquid nitrogen and stored at −80 °C until use.

### 4.2. Identification of ZmWOX Genes

The identification of maize *WOX* genes was performed using lasted assembly genome data (Zm-B73-REFERENCE-NAM-5.0) (download in MaizeGDB, https://maizegdb.org, accessed on 28 September 2023) of inbred line B73. AtWOXs and OsWOXs protein sequences (relative ID shown in Supplementary Table S2) (download in Ensembl Plants, http://plants.ensembl.org, accessed on 28 September 2023) were used as queries to compare with maize genome using BLASTP (package v2.6.0+) (E-value: 1 × 10^−5^ and identity threshold > 50%). The obtained non-redundant protein sequences were further used to refine the selection and identify whether the conserved DNA-binding motif-Homeodomain (HMM, hidden Markov model, login number: PF00046) was present. This was made using searches in the Pfam database (http://pfam-legacy.xfam.org/, accessed on 28 September 2023) [53]. The physical parameters and subcellular localization of maize WOX proteins were determined by the online software ExPASy (https://web.expasy.org/protparam/, accessed on 7 October 2023) and WoLF PSORT (https://wolfpsort.hgc.jp/, accessed on 7 October 2023).

### 4.3. Phylogenetic and Structural Analyses of ZmWOX Genes

The identified maize WOX proteins and reported WOXs in rice, wheat, Sorghum, and Arabidopsis (relative ID shown in Supplementary Tables S1 and S2) [15,16] were aligned with MUSCLE v.5.1.0 [54]. MEGA7 (Version 7.0) was used to build the phylogenetic tree under the neighbor-joining approach (model: Jones, Taylor, and Thornton) with 1000 bootstrap replicates. The conserved motifs of ZmWOXs were analyzed by MEME (Version 5.5.0, parameters: -nmotifs 10) [55] and visualized by TBtools (v1.120) [56]. The exon–intron structures of ZmWOX genes were also visualized with TBtools (v1.120) (default parameters) [56].

### 4.4. Chromosomal Localization and Collinearity Analysis

The chromosomal location information of the *ZmWOXs* was obtained from its genome annotation file in Maize GDB. Using MCScan (Version X, default parameters) [57] to explore the covariance relationships of *WOX* genes in different species, the result of chromosomal localization and collinearity analysis (using one-step MCScanX function) were then visualized by TBtools (v1.120) [56].

### 4.5. Cis-Acting Element Analysis

The 2000 bp promoter sequences upstream of the transcription start site of each *ZmWOX* gene were downloaded from the Maize GDB database (https://maizegdb.org, accessed on 13 November 2023). The online software PLACE (http://bioinformatics.psb.ugent.be/webtools/plantcare/html/, accessed on 17 November 2023) [58] were used to predict possible cis-elements, and the results were visualized by TBtools (v1.120) with default parameters [56].

### 4.6. Transcriptome Data Analysis

The RNA-seq data used for tissue-specific expression analysis were obtained in a published report [40]. The transcriptome sequencing data of maize under heat/cold [43], drought [41], waterlogging [44], and salt [42] stresses were used to explore the *ZmWOX* gene expression patterns under abiotic stress. All RNA-seq data were reanalyzed using the new maize B73 genome information (Section 4.2). Heatmap construction was made according to the fragments per kilobase per million (FPKM) values of sequencing data (2–3 biological replicates were plotted using mean values) and visualized by TBtools (v1.120) [56]. The NCBI RNA-seq data links are shown in Supplementary Tables S4 and S5.

### 4.7. RT-qPCR Analysis of Gene Expression

The total leaf RNA extraction was performed following a published protocol [59]. About 1 μg RNA was used for first-strand cDNA synthesis according to the description in the kit (CWBIO, Beijing, China). The RT-qPCR Mix (20 μL) included 6 μL diluted cDNA, 2 μL RNase-free water, a total of 2 μL each primer, and 10 μL SYBR Mix (Thermo Fisher Scientific, Waltham, MA, USA). PCR was performed on a CFX96 Real-Time System (Bio-Rad, Hercules, CA, USA) following this procedure: 94 °C for 5 min, followed by 43 cycles of 94 °C for 15 s and 60 °C for 25 s. Melting curves were obtained at 65–95 °C in the final step. There are three biological replicates (mixing three independent individuals for one biological replicate) for each sample and two technical replicates (calculation of mean values) for each biological replicate. The transcripts’ relative abundance was calculated by the 2^−∆∆Ct^ method [60]. The *ZmActin1* gene (Zm00001eb348450) was used as the internal control. All primers are listed in Supplementary Table S6.

### 4.8. Subcellular Localization

The CDS of *ZmWOX1* and *ZmWOX18* were amplified using primers ZmWOX1-F/R and ZmWOX18-F/R (Supplementary Table S6) for subcellular localization of ZmWOX1 and ZmWOX18. They were then ligated into the pROKII vector [59]. GFP (Empty pROKII vector) control or ZmWOX1/18-GFP vectors were separately cotransformed with a nuclear localization marker (D53-RFP) into 4-week-old tobacco epidermal cells following a published report [61]. The cells were dark cultured for one day, under 16 h/8 h light/dark at 25 °C for two days. GFP, RFP, and chlorophyll autofluorescence signals (ChI) were observed with a scanning confocal microscope (Andor Revolution WD, Belfast, Northern Ireland, UK).

### 4.9. Transactivation Activity Assays

The CDS of *ZmWOX1* and *ZmWOX18* was amplified using specific primers (ZmWOX1-Y-F/R and ZmWOX18-Y-F/R) listed in Supplementary Table S6, and ligated into pGBKT7, fused with the Gal4-DNA-binding domain (BD-WOX1 and BD-WOX18). The pGADT7 and BD-WOX1/18 vectors were cotransformed into yeast strain Y2HGold. pGBKT7-p53 + pGADT7-largeT and pGBKT7-laminC + pGADT7-largeT were positive and negative controls, respectively. The transformed yeast cells were successively cultured in SD/-Trp/-Leu and SD-Trp/-Leu/-His/-Ade/medium with or without X-α-Gal for four days.

### 4.10. Statistical Analysis

Statistical analysis was performed using SPSS v19.0 (SPSS, Chicago, IL, USA). Significance was assessed using the Student’s *t*-test. One or two asterisks against error bars of histograms are used to indicate means that are statistically different at *p* < 0.05 or *p* < 0.01, respectively. “*n*” indicates the number of independent biological replicates. For RT-qPCR, the results are presented as the mean ± SD (*n* = 3 biological replicates, mixing three independent individuals for one biological replicate).

## 5. Conclusions

This study identified 22 *ZmWOX* genes, and a systematic analysis in maize was performed based on the last B73 genome data. All *ZmWOX* genes can be divided into three groups, and segmental duplication may be the main contribution to *ZmWOX* family expansion. Some *WOX* genes may be evolutionarily conservative in maize, wheat, sorghum, rice, and Arabidopsis. Published transcriptome data analysis showed that, except for *ZmWOX6* and *ZmWOX8*, the expression of other *ZmWOXs* displayed tissue-specific patterns, suggesting that these genes may be involved in maize growth and development. Furthermore, six members of the *ZmWOX* genes, *ZmWOX1*, *4*, *6*, *13*, *16*, and *18*, responded to the abiotic stresses. Meanwhile, ZmWOX1 and ZmWOX18 might play opposite regulatory roles in maize responses to stress. This study provides a reference for the subsequent *ZmWOX* gene function verification study.

## Figures and Tables

**Table 1:** Characteristics of *WOX* genes in maize.

MazieGDB ID	Gene Name	CDS (bp)	Protein Size (aa)	MW (kDa)	pI	Subcellular Location
Zm00001eb015500	*ZmWOX1*	945	314	32.01	6.31	Nucleus
Zm00001eb067310	*ZmWOX2*	963	320	33.09	8.21	Chloroplast
Zm00001eb092480	*ZmWOX3*	789	262	27.84	8.37	Nucleus
Zm00001eb147630	*ZmWOX4*	666	221	24.76	8.36	Nucleus
Zm00001eb148390	*ZmWOX5*	975	324	34.81	9.16	Nucleus
Zm00001eb149680	*ZmWOX6*	822	273	30.39	6.06	Nucleus
Zm00001eb157360	*ZmWOX7*	1551	516	52.90	7.15	Nucleus
Zm00001eb180280	*ZmWOX8*	252	83	10.01	10.52	Cytosol/Nucleus
Zm00001eb197430	*ZmWOX9*	399	132	14.92	9.85	Mitochondrion
Zm00001eb265710	*ZmWOX10*	777	258	27.47	7.13	Nucleus
Zm00001eb280440	*ZmWOX11*	381	126	14.26	9.55	Nucleus
Zm00001eb295920	*ZmWOX12*	1518	505	53.26	7.21	Nucleus
Zm00001eb330990	*ZmWOX13*	780	259	27.52	7.02	Nucleus
Zm00001eb355310	*ZmWOX14*	525	174	19.38	9.60	Nucleus
Zm00001eb359810	*ZmWOX15*	1545	514	53.24	7.27	Mitochondrion
Zm00001eb367200	*ZmWOX16*	708	235	26.47	9.72	Nucleus
Zm00001eb367990	*ZmWOX17*	1026	341	36.77	9.18	Nucleus
Zm00001eb368970	*ZmWOX18*	849	282	30.98	6.46	Nucleus
Zm00001eb395430	*ZmWOX19*	948	315	32.41	6.84	Nucleus
Zm00001eb414580	*ZmWOX20*	885	294	31.37	7.91	Nucleus
Zm00001eb432140	*ZmWOX21*	753	250	27.72	8.63	Chloroplast
Zm00001eb433010	*ZmWOX22*	978	325	33.17	5.56	Chloroplast

## Data Availability

The data presented in this study are available on request from the corresponding author.

## Supplementary Materials

The following supporting information can be downloaded at https://www.mdpi.com/article/10.3390/ijms25010441/s1.
