# Supplementary material for: A Comprehensive Identification and Expression Analysis of the WUSCHEL Homeobox-Containing Protein Family Reveals Their Special Role in Development and Abiotic Stress Response in Zea mays L
Source: Int J Mol Sci. 2023 Dec 28;25(1):441. doi: 10.3390/ijms25010441 (PMC10779079; doi:10.3390/ijms25010441)
Supplement: Supplementary file 1 [file ijms-25-00441-s001.zip › ijms-2791777-supplementary-final/Supplementary Table S1.docx]

| Supplementary Table S1. Chromosome location of *WOX* genes in maize and sorghum. | | |
| --- | --- | --- |
| **MaizeGDB ID** | **Gene name** | **Chromosome location** |
|  | **Mazie** | chr1: 53036735...53043529  chr2: 3677807...3682657  chr2: 146773635...146778130  chr3: 183346657...183351224  chr3: 186156066...186162456  chr3: 190588267...190596563  chr3: 216168490...216176523  chr4: 92675188...92678770  chr4: 196232087...196235814  chr6: 37924213...37929351  chr6: 127211873...127215253  chr6: 176083028...176090643  chr7: 182791742...182798613  chr8: 135945836...135949777  chr8: 153204861...153211579  chr8: 173812943...173817004  chr8: 175427986...175434603  chr8: 177185124...177191371  chr9: 139044438...139051343  chr10: 71362450...71367062  chr10: 147565228...147569965  chr10: 149308612...149313271  chr1: 67421476...67424954  chr2: 76925288…76927871  chr3: 58843301…58847815  chr3: 65980404…65983717  chr3: 67043467…67046724  chr3: 67781814…67783428  chr5: 3893891…3895467  chr6: 58182939…58184142  chr6: 59228238…59230492  chr7: 11950505...11952046  chr9: 2106899...2108852  chr9: 57264989...57269005 |
| Zm00001eb015500 | *ZmWOX1* |  |
| Zm00001eb067310 | *ZmWOX2* |  |
| Zm00001eb092480 | *ZmWOX3* |  |
| Zm00001eb147630 | *ZmWOX4* |  |
| Zm00001eb148390 | *ZmWOX5* |  |
| Zm00001eb149680  Zm00001eb157360  Zm00001eb180280  Zm00001eb197430  Zm00001eb265710  Zm00001eb280440  Zm00001eb295920  Zm00001eb330990  Zm00001eb355310  Zm00001eb359810  Zm00001eb367200  Zm00001eb367990  Zm00001eb368970  Zm00001eb395430  Zm00001eb414580  Zm00001eb432140  Zm00001eb433010  **NCBI ID**  XM_002465290.2  XM_021452828.1  XM_021457904.1  XM_002456495.2  XM_002458694.2  XM_002458736.2  XM_002448974.2  XM_002448595.2  XM_021464099.1  XM_021465477.1  XM_021447076.1  XM_002440209.2 | *ZmWOX6*  *ZmWOX7*  *ZmWOX8*  *ZmWOX9*  *ZmWOX10*  *ZmWOX11*  *ZmWOX12*  *ZmWOX13*  *ZmWOX14*  *ZmWOX15*  *ZmWOX16*  *ZmWOX17*  *ZmWOX18*  *ZmWOX19*  *ZmWOX20*  *ZmWOX21*  *ZmWOX22*  **Sorghum**  *SbWOX1*  *SbWOX2*  *SbWOX3*  *SbWOX4*  *SbWOX5*  *SbWOX6*  *SbWOX7*  *SbWOX8*  *SbWOX9*  *SbWOX10*  *SbWOX11*  *SbWOX12* |  |
