# Supplementary material for: A Comprehensive Identification and Expression Analysis of the WUSCHEL Homeobox-Containing Protein Family Reveals Their Special Role in Development and Abiotic Stress Response in Zea mays L
Source: Int J Mol Sci. 2023 Dec 28;25(1):441. doi: 10.3390/ijms25010441 (PMC10779079; doi:10.3390/ijms25010441)
Supplement: Supplementary file 1 [file ijms-25-00441-s001.zip › ijms-2791777-supplementary-final/Supplementary Table S2.docx]

**Supplementary Table S2**: Summary of the *AtWOX*, *OsWOX* and *TaWOX* gene family

| **Name** | **Ensemble Tanscript ID** |
| --- | --- |
| OsWOX1 | Os04g0663600 |
| OsWOX2 | Os05g0118700 |
| OsWOX3 | Os12g0101600 |
| OsWOX4 | Os04g0649400 |
| OsWOX5 | Os01g0840300 |
| OsWOX6 | Os03g0325600 |
| OsWOX7 | Os01g0667400 |
| OsWOX8 | Os01g0818400 |
| OsWOX9 | Os01g0854500 |
| OsWOX10 | Os08g0242400 |
| OsWOX11 | Os07g0684900 |
| OsWOX12 | Os05g0564500 |
| OsWOX13 | Os01g0667400 |
| AtWUS | AT2G17950 |
| AtWOX1 | AT3G18010 |
| AtWOX2 | AT5G59340 |
| AtWOX3 | AT2G28610 |
| AtWOX4 | AT1G46480 |
| AtWOX5 | AT3G11260 |
| AtWOX6 | AT2G01500 |
| AtWOX7 | AT5G05770 |
| AtWOX8 | AT5G45980 |
| AtWOX9 | AT2G33880 |
| AtWOX10 | AT1G20710 |
| AtWOX11 | AT3G03660 |
| AtWOX12 | AT5G17810 |
| AtWOX13 | AT4G35550 |
| AtWOX14 | AT1G20700 |
| TaWUSa | TraesCS2A02G491900.1 |
| TaWOX2a | TraesCS1A02G052000.1 |
| TaWOX3a | TraesCS5A02G157300.1 |
| TaWOX4a | TraesCS2A02G514000.1 |
| TaWOX5a | TraesCS5A02G085000.1 |
| TaWOX6a | TraesCS4A02G130200.1 |
| TaWOX7a | TraesCS3A02G247200.1 |
| TaWOX8a | TraesCS3A02G341700.1 |
| TaWOX9a | TraesCS3A02G368100.1 |
| TaWOX10a | TraesCS3A02G073500.1 |
| TaWOX11a | TraesCS2A02G100700.1 |
| TaWOX12a | TraesCS1A02G399400.1 |
| TaWOX13a | TraesCS3A02G358100.1 |
| TaWOX14b | TraesCS3B02G391200.1 |
