# Supplementary material for: A Comprehensive Identification and Expression Analysis of the WUSCHEL Homeobox-Containing Protein Family Reveals Their Special Role in Development and Abiotic Stress Response in Zea mays L
Source: Int J Mol Sci. 2023 Dec 28;25(1):441. doi: 10.3390/ijms25010441 (PMC10779079; doi:10.3390/ijms25010441)
Supplement: Supplementary file 1 [file ijms-25-00441-s001.zip › ijms-2791777-supplementary-final/Supplementary Table S6.pdf]

Table S6. List of Primers

Primer name	Primer sequence
ZmActin1-F	TACGAGATGCCTGATGGTCAGGTCA
ZmActin1-R	TGGAGTTGTACGTGGCCTCATGGAC
ZmWOX1-RT-F	ACGTGATGCTCGTCCATTC
ZmWOX1-RT-R	CTAGCTACTTGCGGCAGTTT
ZmWOX4-RT-F	CCGGAAGCAACCAAATCAG
ZmWOX4-RT-R	GCTACACTACACCGCCAGTTAAG
ZmWOX6-RT-F	CAGGCCGCTTCTTTACCG
ZmWOX6-RT-R	CGCCCATAGCCTTGTTGTC
ZmWOX13-RT-F	CAGCAGCAGCTTTACTACTCGC
ZmWOX13-RT-R	CCCGAACATGGACCGCAG
ZmWOX16-RT-F	AAAAGCAAACGGGAGGCC
ZmWOX16-RT-R	CTCCTGCTGCTCCACAACG
ZmWOX18-RT-F	GCTCGGAGGATACGTCAAG
ZmWOX18-RT-R	GCAATGGTGTCCTGGTGC
ZmWOX1-F	AGAACACGGGGGACTCTAGAATGGAGGGGGGCAGCAA
ZmWOX1-R	CTCACCATGGTACCCTCGAGAGTTGGCCTTGCTACCAAGAAA
ZmWOX1-Y-F	CCTGCATATGGCCATGGAGGCC ATGGAGGGGGGCAGCAA
	TATGCGGCCGCTGCAGGTCGAC
ZmWOX1-Y-R	TTAAGTTGGCCTTGCTACCAAG
	AGAACACGGGGGACTCTAGAATGGACTGGGGGAACAGG
ZmWOX18-F	CTCACCATGGTACCCTCGAG TCCAAACATGCCAAAGCC
ZmWOX18-R	CCTGCATATGGCCATGGAGGCC
ZmWOX18-Y-F	ATGGACTGGGGGAACAGGAC
	TATGCGGCCGCTGCAGGTCGAC
ZmWOX18-Y-R	TCATCCAAACATGCCAAAGC
