# Supplementary figures and images for: A Comprehensive Identification and Expression Analysis of the WUSCHEL Homeobox-Containing Protein Family Reveals Their Special Role in Development and Abiotic Stress Response in Zea mays L
Source: Int J Mol Sci. 2023 Dec 28;25(1):441. doi: 10.3390/ijms25010441 (PMC10779079; doi:10.3390/ijms25010441)

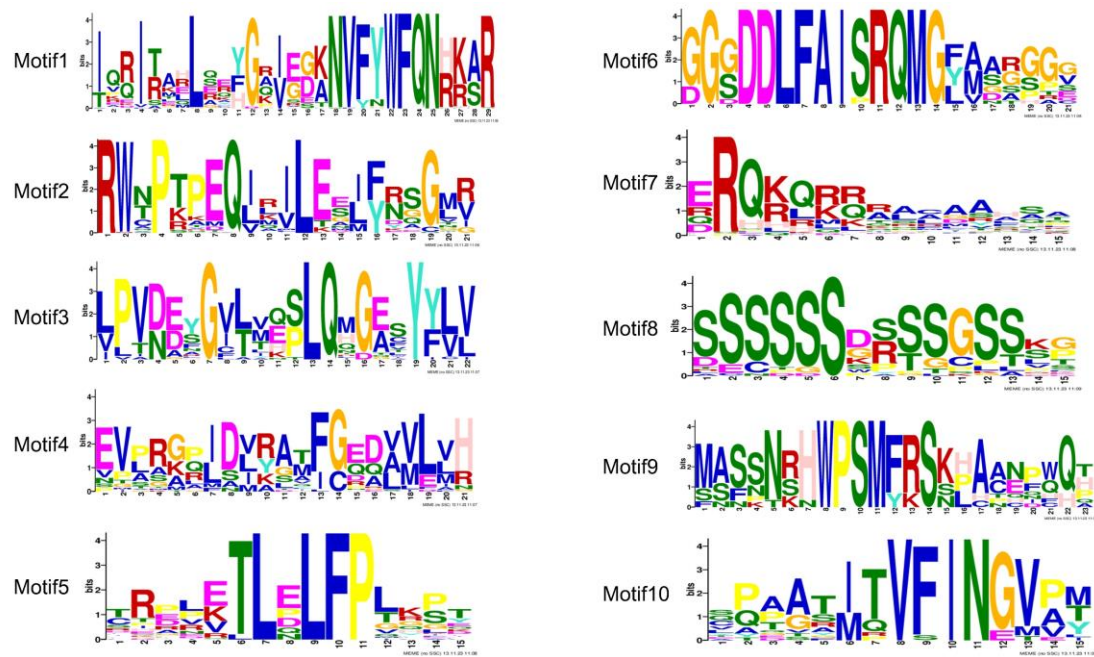

**Figure S1.** The common sequence of ten conserved motifs listed in Figure 2.

Supplement: Supplementary file 1 [file ijms-25-00441-s001.zip › ijms-2791777-supplementary-final/Figure S1.pdf]
